# Clevidipine infusion for blood pressure management after successful revascularisation in acute ischaemic stroke: the CLEVER study

**DOI:** 10.1093/esj/aakag005

**Published:** 2026-02-17

**Authors:** Mouhammad A Jumaa, Khaled Gharaibeh, Richard E Burgess, Rahul Rao, Marion J Oliver, Adam Mierzwa, Hisham S Alhajala, Ashan Ali, Ashutosh P Jadhav, Alicia C Castonguay, Hijrah El-Sabae, Syed F Zaidi

**Affiliations:** Department of Neurology, ProMedica Toledo Hospital, Toledo, OH, United States; Department of Neurology, University of Toledo, Toledo, OH, United States; Department of Neurology, University of Toledo, Toledo, OH, United States; Department of Neurology, University of Toledo, Toledo, OH, United States; Department of Neurology, University of Toledo, Toledo, OH, United States; Department of Neurology, Advocate Aurora Health, Park Ridge, IL, United States; Department of Neurology, University of Toledo, Toledo, OH, United States; Department of Neurology, University of Toledo, Toledo, OH, United States; Department of Neurology, University of Toledo, Toledo, OH, United States; Department of Neurology, Barrow Neurological Institute, Phoenix, AZ, United States; Department of Neurology, ProMedica Toledo Hospital, Toledo, OH, United States; HAE MedSci Solutions, Irvine, CA, United States; Department of Neurology, ProMedica Toledo Hospital, Toledo, OH, United States; Department of Neurology, University of Toledo, Toledo, OH, United States

**Keywords:** blood pressure, management, occlusion, stroke, thrombectomy

## Abstract

**Background:**

Limited studies have provided guidance on the optimal systolic blood pressure (SBP) after mechanical thrombectomy (MT) in acute ischemic stroke patients. In the Clevidipine Infusion for Blood Pressure Management After Successful Revascularization in Acute Ischemic Stroke (CLEVER) trial, our aim was to study the safety and efficacy of intensive SBP control with a Clevidipine infusion as the first-line agent.

**Methods:**

The CLEVER trial was an investigator-initiated, single-center, open-label, randomized, controlled trial. Patients were randomized 1:1 to a SBP goal after successful MT (mTICI 2c or greater) of either 90-120mmHg (Intensive BP Management) or 90-160mmHg (Standard BP Management). The primary outcomes were time to target blood pressure and incidence of any hemorrhagic conversion at 24 hours.

**Results:**

Between October 2021 and December 2023, 80 eligible patients were enrolled, 40 each into the intensive BP and the standard BP management cohorts. Overall, 72% of all BP measurements in the intensive BP management group were within the target range, compared to 93% in the standard BP management group (*p*<0.001). The median time from the initiation of Clevidipine infusion until reaching the target SBP was significantly shorter in the standard BP management group, 10 minutes [IQR 5.0-45.0] versus 20 IQR 12.5-42.5] (95%CI:5.0-20.0, *p*=0.002). The incidence of hemorrhagic transformation per core lab was not significantly different in the intensive BP management group (32.5%) and the standard BP management group (35.0%); adjusted OR (0.93 [95%CI, 0.30-2.85]; *p*=0.89).

**Conclusion:**

In the randomised CLEVER trial, intensive BP control using clevidipine after MT failed to reduce the rate of haemorrhagic conversion or sICH and resulted in a numerically lower rate of good clinical outcome compared to standard BP control. Clevidipine was well tolerated in both cohorts and demonstrated a similar safety profile. Larger studies are needed to understand the efficacy and safety of clevidipine for BP control and the optimal BP threshold after MT.

## Graphical Abstract

**Graphical Abstract:**
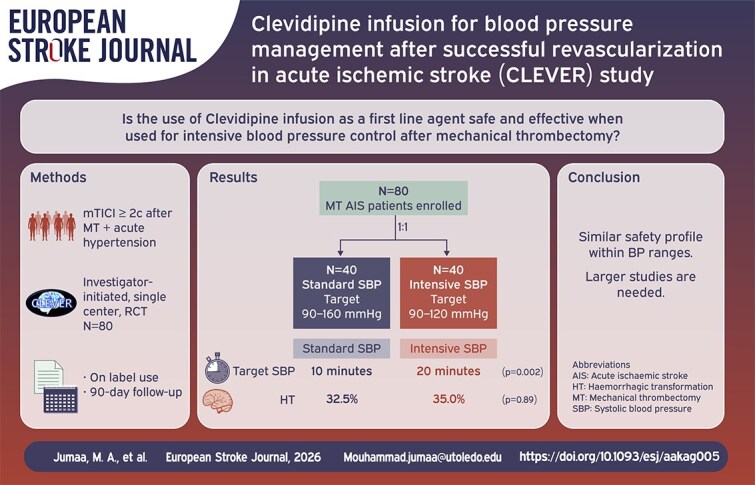

## Introduction

Post mechanical thrombectomy (MT) blood pressure (BP) management remains heterogeneous among practitioners when managing LVO ischaemic strokes.^1^ While an upper BP threshold may preserve blood vessel wall integrity and prevent reperfusion injury and haemorrhagic transformation, a lower limit ensures adequate perfusion to the stroke penumbra permitting cell survival.^1-6^ Several factors may influence an appropriate BP range including LVO mechanism, thrombolysis administration, total occlusion time, pre-procedural infarction area, MT technique and most importantly, final modified thrombolysis in cerebral infarction (mTICI) score.^1,2,7-9^ Yet, a deficiency in impactful studies limited the current American Stroke Association/American Heart Association (ASA/AHA) guideline to only endorse a 180/105 mmHg BP ceiling (COR 2A, LOE B-NR) resulting in wide practice variability across thrombectomy centres.^8^

Recent trials have attempted to shed light on BP management after MT and provide further evidence to guide proceduralists. For example, the Intensive Blood Pressure Control after Endovascular Thrombectomy for Acute Ischaemic Stroke (ENCHANTED2) and Intensive vs Conventional Blood Pressure Lowering after Endovascular Thrombectomy in Acute Ischaemic Stroke (OPTIMAL-BP) analysed a population with proportionately higher intracranial atherosclerotic disease and concluded that intensive BP targets portends diminished functional independence without a mortality benefit.^10,11^ However, 2 other randomised controlled trials with an embolic LVO population failed to demonstrate any benefit or harm when applying parameters with a lower BP target range.^12,13^

Despite invasive haemodynamic monitoring in the intensive care setting, BP variability remains common and may result in worse outcomes.^14^ In such instances, further intricacies focus on intravenous antihypertensive agents. Clevidipine, a dihydropyridine calcium channel blocker, has gained traction due to its enhanced ability to achieve and maintain BP targets.^15,16^ Early cardiothoracic surgery literature demonstrated the superiority of clevidipine over placebo and standard methods of controlling BP.^17-19^ Later, clevidipine attained haemodynamic stability in intracranial haemorrhage, subarachnoid haemorrhage and ischaemic stroke without significant BP variability or “overshooting” beyond the minimum BP range.^20-23^

In the Clevidipine Infusion for Blood Pressure Management After Successful Revascularisation in Acute Ischaemic Stroke (CLEVER) trial, our aim was to study the safety and efficacy of intensive systolic blood pressure (SBP) control with clevidipine infusion as the first-line agent.

## Patients and methods

### Trial design

The authors declare that all supporting data are available within the article [and its Online Supplementary Files]. The CLEVER trial was an investigator-initiated, single-centre, open-label, randomised, controlled trial to assess the safety and efficacy of intensive BP control using clevidipine for BP management in AIS patients undergoing standard of care MT within 24 h of symptoms onset. Clevidipine was administered per US Food and Drug Administration (FDA) approved on-label use. Patients enrolled into CLEVER were randomised 1:1 to a SBP goal after successful MT (mTICI 2c or greater) of either 90–120 (intensive BP management) or 90–160 mmHg (standard BP management) and were followed up to 3 months after clevidipine treatment. An independent imaging core lab assessed final angiographic data points and 24-h imaging for infarct volume and haemorrhagic transformation per SITS-MOST criteria. The trial was registered on clinicaltrials.gov: NCT05175547. The study sponsor (Chiesi USA, Inc.) had no role in the design, analysis or writing of the manuscript.

### Eligibility

Patients were considered for the CLEVER study if the following key inclusion criteria were met: (1) age ≥ 18 years, (2) acute hypertension (SBP of greater than 130 mmHg) at recanalisation, (3) anterior circulation ischaemic stroke symptoms and confirmed occlusion (internal carotid artery [ICA], M1 or M2) on angiogram with MT initiated within 24 h since last known well, (4) successful revascularisation score of mTICI 2c or higher after MT and (5) signed informed consent within 30 min from end of MT procedure. Key exclusion criteria included, (1) presence of any haemorrhage and/or ASPECT score ≤ 6 on baseline head CT scan, (2) acute traumatic brain injury, (3) acute or recent ST-segment elevation myocardial infarction in the last 30 days, (4) severe arrhythmias, unstable cardiac function and (5) intracranial atherosclerosis (culprit lesion or diffuse moderate to severe). Enrollment of tandem occlusions, defined as an extracranial ICA occlusion with occlusion in the ICA, M1 or M2 vessel, was allowed in CLEVER. A complete list of inclusion and exclusion criteria is listed in Table SI. Patients were considered enrolled into CLEVER when the patient or their legally authorised representative signed the informed consent form, all eligibility requirements were met and the patient was randomised into the study.

### Intervention

**Figure 1:**
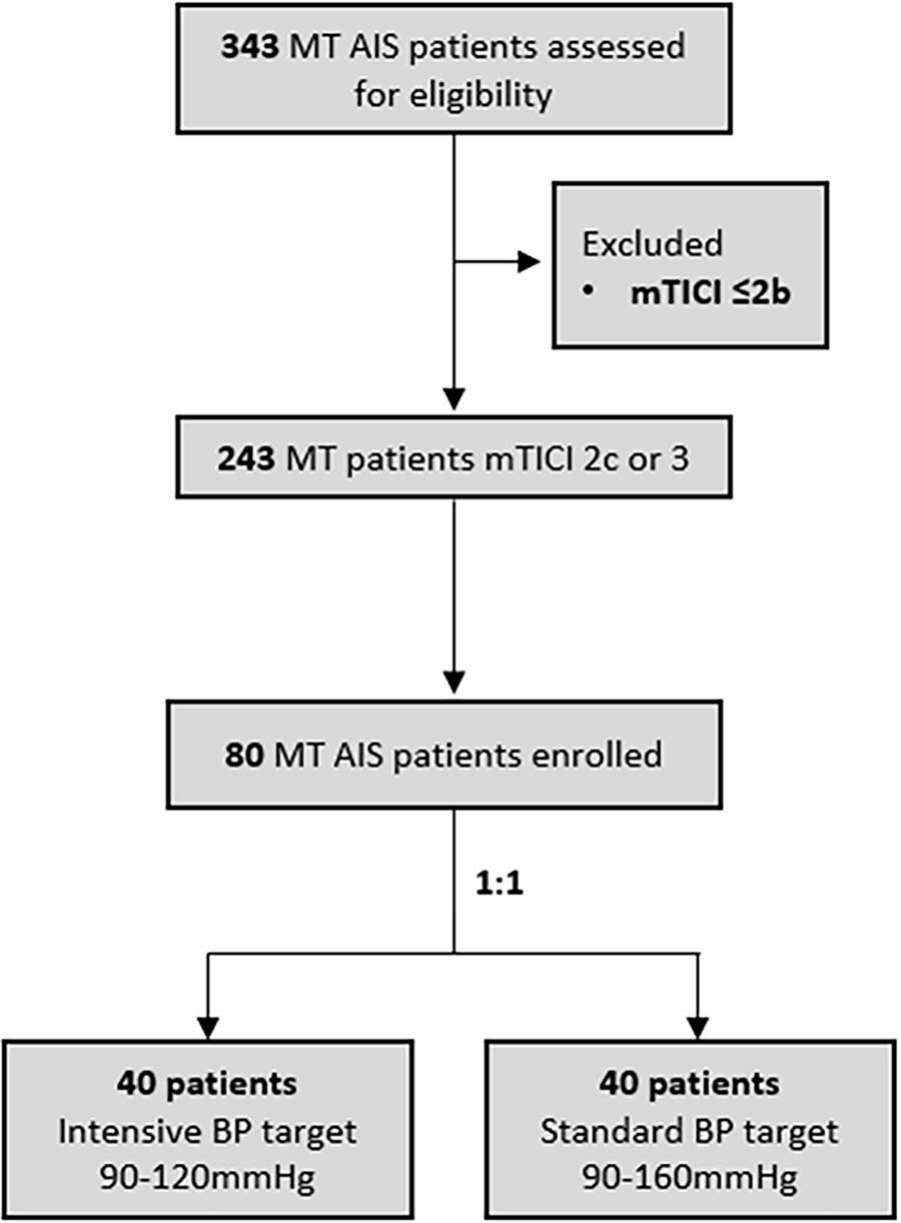
CLEVER trial flow chart. Abbreviation: CLEVER, Clevidipine Infusion for Blood Pressure Management After Successful Revascularisation in Acute Ischaemic Stroke.

At the end of the MT procedure, patients were randomised 1:1 to a target BP of 90–120 or 90–160 mmHg (Figure 1). Intravenous clevidipine was administered per FDA approved on-label indication. Local standard of care protocols were followed to administer clevidipine. Per on-label use, the initial dose of clevidipine was 1–2 mg/h and the dosage was titrated per individualised response of each patient and respective BP goal. Blood pressure was measured throughout the infusion and up to 24 h with an automated BP monitor and according to the BP monitoring protocol from the ATACH-II trial.^24^ Blood pressure measurements were taken every 5 min for the first 15 min after clevidipine was started, then every 15 min for the remainder of the first hour unless the dose was adjusted (then every 5 min for 15 min during dose adjustments). After maintenance infusion, BP measurements were taken every 30 min until SBP was increased by 10 mmHg over the SBP measurement at the end of infusion, alternate antihypertensive therapy was initiated or a maximum of 24 h has elapsed. Additional hypertensive agents were permitted if clevidipine was maximised per AHA guidelines.

### Endpoints

The primary efficacy endpoint of this study was the time to achieve the target SBP, defined as the time from clevidipine initiation to target SBP. The primary safety endpoint was the incidence of any haemorrhagic conversion at 24 h as adjudicated by an independent imaging core lab. Secondary endpoints were the efficacy of clevidipine in maintaining BP within range using area under the curve analysis of BP excursions beyond predetermined upper and lower limits using statistical models from the ECLIPSE trials, delayed ICH after 24 h, mRS 0–2 or return to baseline at 90 days and mortality rate at 90 days. Safety assessments included measures of incidence of any haemorrhagic conversion at 24 h according to the Heidelberg Bleeding Classification criteria.^25^

### Statistical analysis

#### SBP descriptive analysis

The mean SBP values over the initial 24 h were compared between the standard and intensive target groups. Furthermore, SBP variability between the groups was compared using 5 well-characterised statistical methods, as follows: SD, coefficient of variation (CV), average real variability (ARV), successive variation (SV) and residual SD (rSD). To account for the effects of age, mean SBP, baseline ASPECTS and initial NIHSS on SBP variability, adjusted SBP variability values were computed as the residuals from separate regression models that adjusted each SBP variability metric (SD, CV, ARV, SV and rSD) for the above-mentioned variables.^26^ To evaluate whether patients remained in their assigned groups, the proportion of patients who switched their SBP target goals and switching occurrence over the 24-h period following reperfusion was described. To explore whether the SBP varied over time in patients after they reached their initial target group, a linear mixed-effects model was employed with SBP as the dependent variable, a fixed effect of time and a patient-specific random effect to account for the multiple observations per patient. To quantify the difference in SBP between groups, adjusted overall difference between groups was calculated using a linear mixed model with a fixed effect of treatment group, a fixed effect of time, a fixed interaction between treatment and time and a patient-specific random effect.^11^ All *P* values were 2-sided but not adjusted for multiple analyses. Statistical significance level was defined at α = 0.05. All analyses were conducted using R software (R Foundation for Statistical Computing).

#### Baseline characteristics and endpoints

Continuous variables were summarised using descriptive statistics. Categorical variables were presented using frequencies and percentages. This pilot study was designed without formal power calculation due to lack of prospective data at the time of the initial design. If applicable, CIs were provided with descriptive statistics to assess the precision of estimates.

#### Sensitivity analysis

Variables with *P* values less than .2 on the univariate analysis as well as selected clinically significant variables were included into the sensitivity subgroup analysis. Interaction terms were included in the final model and summarised in the Supplementary Material.

## Results

Between October 2021 and December 2023, 80 eligible patients were consented and enrolled into the CLEVER trial, with 40 patients randomised into the intensive BP management cohort and 40 patients to the standard BP management cohort (Figure 1).

### Baseline characteristics

Baseline demographic characteristics of patients were balanced between treatment groups (Table 1). Mean age was 74.5 [62.75–81.5] and 71.5 [64.0–82.25] years in the intensive BP and standard BP management groups, respectively. Over half of the population was female and white in both cohorts. Most patients had a history of hypertension (80% vs 87.5%) and hyperlipidemia (70% vs 77.5%) in the intensive BP management and standard BP management groups, respectively. Median ASPECTS (10 [IQR 9–10]) was similar in the cohorts. Intravenous thrombolysis was administered in 50% of intensive BP management patients and 45% of standard BP management patients. Most occlusions were in the MCA (73.8%), with 52.5% and 32.5% M1 and 20% and 42.5% M2 in the intensive BP management and standard BP management groups, respectively. Cardioembolic stroke was the most common stroke aetiology in both groups. Median onset to arrival time was similar in both groups (220.5 [IQR 168.75–384.25] vs 284 [IQR 203–629.8] min, *P* = .11).

**Table 1:** Baseline, clinical and angiographic characteristics of CLEVER patients

**Characteristic**	**Intensive BP group (*n* = 40)**	**Standard BP group (*n* = 40)**	***P*-value**
**Pre-stroke mRS (%, *n*)**			.55
0	28	22	
1	4	6	
2	6	8	
3	2	2	
4	0	2	
**Medications**			
Antihypertensive drugs	11 (28.9%)	7 (17.9%)	.29
Statin or other lipid lowering drug	18 (45%)	23 (57.5%)	.37
Aspirin or other antiplatelet drug	15 (37.5%)	16 (40.0%)	1.00
Anticoagulation drug	10 (25%)	12 (30%)	.80
Baseline NIHSS (median, IQR)	16.5 [11.75–21.25]	15.5 [11.75–21.0]	.92
IV thrombolysis (%, *n*)	20 (50%)	18 (45%)	.82
ASPECTS (median, IQR)	10 [9–10]	10 [9–10]	.24
Transfer (%, *n*)	30 (75%)	27 (67.5%)	.62
**TOAST classification**			.89
Cardioembolism	24 (60.0%)	23 (57.5%)	
Large vessel atherosclerosis	3 (7.5%)	5 (12.5%)	
Stroke of other determined etiology	3 (7.5%)	2 (5.0%)	
Undetermined, negative evaluation	10 (25.0%)	10 (25.0%)	
**Primary occlusion site**			.18
M1	22	16	
M2	8	16	
ICA-T	9	8	
Tandem	2	2	1.0
Onset to arrival (min) (median, IQR)	220.5 [168.8–384.3]	284.0 [203.0–629.8]	.11
Onset to revascularisation (min) (median, IQR)	273.5 [209.3–416.3]	339.0 [225.5–664.3]	.33
**Final revascularisation grade (mTICI)**			.18
2c	16 (40.0%)	23 (57.5%)	
3	24 (60.0%)	17 (42.5%)	
SBP at recanalisation (mean, SD)	157.4 (26.6)	157.9 (23.0)	.92
DBP at recanalisation (mean, SD)	79.6 (16.9)	85.5 (19.6)	.15
**Technique**			1.0
Aspiration (%, *n*)	29 (72.5%)	28 (70.0%)	
Aspiration and stent retriever	11 (27.5%)	12 (30.0%)	
Number of passes (median, IQR)	2 [1–3]	2 [1–3]	.92

Abbreviations: CLEVER = Clevidipine Infusion for Blood Pressure Management After Successful Revascularisation in Acute Ischaemic Stroke; DBP = diastolic blood pressure; ICA-T = internal carotid artery terminus; mTICI = modified thrombolysis in cerebral infarction; OR = odds ratio; SBP = systolic blood pressure.

### Procedural characteristics and angiographic outcomes

Median time from onset to randomisation was 273.5 [IQR 209.3–416.3] vs 339.0 [IQR 225.5–664.3] min (*P* = .33) (Table 1). Final mTICI grade was 2c in 40% and 57.5% and 3 in 60% and 42.5% in the intensive BP and standard BP management groups, respectively. The primary MT technique for most patients was aspiration alone (72.5% vs 70%). The mean SBP at recanalisation was 157.4 ± 26.6 mmHg in the intensive BP management group and 157.9 ± 23 mmHg in the standard BP management group.

### Clevidipine infusion and BP measurements

**Figure 2:**
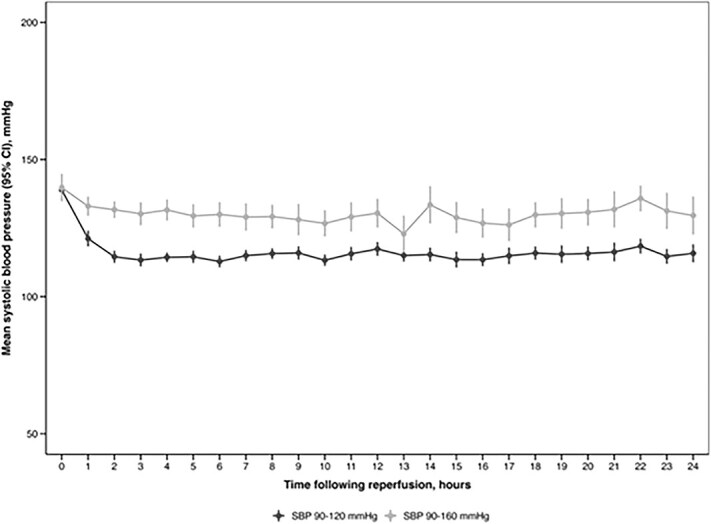
Mean systolic blood pressure over the initial 24 h in both groups.

Figure 2 shows the mean SBP over the initial 24 h in the CLEVER cohorts (Figure SI). Overall, 72% of all BP measurements in the intensive BP management group were within the target range, compared to 93% in the standard BP management group (*P* < .001). The overall mean SBP was 131 ± 20 mmHg in the standard BP group and 117 ± 15 mmHg in the intensive BP group. The adjusted overall difference in SBP over 24 h between groups was −13.6 mmHg [95%CI, −18.7, −8.6; *P* < .0001]. The SBP changed by a rate of −0.02 mmHg/h (95%CI, −0.08, 0.04; *P* = .51) over the first 24 h. Adjusted SBP SD, COV, SV, rSD and ARV did not significantly differ between the 2 groups (Figure SII).

Overall, 26.2% of recorded BPs were outside the target range over 24 h (Figure SIII). When stratified by treatment group, 30.8% of SBP reads in the standard BP group fell into the intensive BP parameters, while 23.1% in the intensive BP group were outside the intended range.

Moreover, the need for supplementary antihypertensive medications was notably higher among those receiving intensive BP treatment, with 16 (40%) patients requiring adjunctive therapy, whereas, in the standard BP treatment group, only 1 patient (2.5%) required additional medication.

#### Primary efficacy and safety outcome

In the comparison between standard BP and intensive BP management groups, the median time from the initiation of clevidipine infusion until reaching the target SBP differed significantly. In the standard BP group, the median duration was 10 min [IQR 5.0–45.0], while in the intensive BP group, it extended to a median of 20 [IQR 12.5–42.5]. The median time difference was 10 min (95%CI, 5.0–20.0; *P* = .002) (Table 2).

**Table 2:** Primary and secondary in the CLEVER study

	**Intensive group** **(*n* = 40)**	**Standard group** **(*n* = 40)**	**Adjusted OR (95%CI)**	***P*-value**
**Primary outcome**				
Median time infusion to target SBP, min	20 [12.5–42.5]	10 [5.0–45.0]	5.0–20.0	.002
**Secondary outcomes**				
Functional independence at 3 months (mRS 0–2) or return to baseline	19 (47.5%)	23 (57.5%)	1.80 (0.42–7.8)	.43
Excellent outcome (mRS 0–1)	7 (17.5%)	13 (32.5%)	3.01 (0.45–20.1)	.26
Other BP med used	10 (25%)	8 (20%)	0.93 (0.25–3.50)	.92
Median duration of hospitalisation (IQR), days	7 [4–9]	5 [3.75–9]	–	.46
**Safety outcomes**				
Mortality within 3 mo no. (%)	3 (7.5%)	4 (10%)	0.49 (0.045–5.29)	.56
Any haemorrhagic transformation no. (%)	13 (32.5%)	14 (35%)	0.93 (0.30–2.85)	.89
sICH no. (%)	0	1 (2.5%)	–	–
Transient hypotensive events no.	3	4	–	–
Hypotensive events requiring intervention no.	0	1	–	–
Mortality within 3 mo no. (%)	3 (7.5%)	4 (10%)	0.49 (0.045–5.29)	.56
Any haemorrhagic transformation no. (%)	13 (32.5%)	14 (35%)	0.93 (0.30–2.85)	.89

Abbreviations: BP = blood pressure; CLEVER = Clevidipine Infusion for Blood Pressure Management After Successful Revascularisation in Acute Ischaemic Stroke; OR = odds ratio; SBP = systolic blood pressure.

The incidence of haemorrhagic transformation per core lab assessment was not significantly different between the intensive BP group (32.5%) and the standard BP group (35.0%); adjusted odds ratio (OR 0.93 [95%CI, 0.30–2.85]; *P* = .89) (Table 2).

A sensitivity analysis showed no significant differences on the primary outcome, with atrial fibrillation the only variable approaching statistical significance, with atrial fibrillation positive patients demonstrating an OR of 3.97 (95%CI, 1.01–18.2; *P* = .058) (Table SII).

#### Secondary outcomes

There was no statistical significance observed in functional independence, as measured by mRS score of 0–2, between the 2 groups, with an adjusted OR of 1.80 (95%CI, 0.42–7.8; *P* = .43) (Table 2). Similarly, when assessing for an excellent outcome (mRS score of 0–1 at the 3-month follow-up), there remained no statistical significance between the groups, adjusted OR 3.01 (95%CI, 0.45–20.1; *P* = .26).

No statistically significant difference in all-cause mortality within 3 months was observed between the 2 cohorts (adjusted OR 0.49 [95%CI, 0.045–5.29]; *P* = .56) (Table 2). Only 1 patient in the standard management group had sICH. Transient hypotensive events occurred in 3 (7.5%) in the intensive BP group and 4 (10%) in the standard BP group. Only 1 patient had hypotensive events requiring intervention in the intensive BP group. Additionally, transient acute kidney injury (AKI) occurred in 8 (20%) in the intensive BP group and 2 (5%) in the standard BP group. No patients in this study had AKI events requiring intervention. One patient in the intensive BP group experienced pulmonary oedema that required medical treatment on day 2 post-thrombectomy.

Analysis of BP excursions for both cohorts showed that the mean number of excursions (time/magnitude outside limits) was 0.83 ± 3.2 and 0.08 ± 0.7 in the standard BP and the intensive BP management groups, respectively (Table SIII).

## Discussion

In this single-centre, randomised, controlled trial, clevidipine demonstrated notable efficacy and safety in limiting SBP fluctuations in both the intensive and standard BP management cohorts after MT. However, intensive BP control failed to reduce the rate of haemorrhagic conversion or sICH and resulted in a numerically lower rate of good clinical outcome compared to standard BP management.

Clevidipine Infusion for Blood Pressure Management After Successful Revascularisation in Acute Ischaemic Stroke demonstrated a positive trend in functional independence favouring the standard BP management group, which is consistent with the findings of a recent meta-analysis of 4 randomised controlled trials.^14^ Gharaibeh et al. reported a lower likelihood of functional independence (mRS 0–2; OR 0.68; 95%CI, 0.51–0.91; *P* = .009) in the intensive treatment group compared to the conventional treatment cohort. A significant divergence between our study and previous trials lies in the choice of antihypertensive agents. Nicardipine was consistently employed in 3 of the trials referenced in the meta-analysis (OPTIMAL-BP, BP-TARGET and BEST II), while urapidil was favoured in the fourth trial (ENCHANTED-II).^10-13^ Clevidipine Infusion for Blood Pressure Management After Successful Revascularisation in Acute Ischaemic Stroke, in contrast, had a distinctive approach utilising clevidipine as a first-line antihypertensive agent within the initial 24-h post-MT period.

Clevidipine has a fast onset of action, typically 2–4 min and a short half-life (about 1 min), making it an ideal drug for ischaemic stroke patients after thrombectomy when strict BP parameters are warranted. Several studies have demonstrated clevidipine’s ability to achieve target BP quickly, with Levy et al. reporting a median of 6.0 min in pre-operative cardiac patients and Singla et al. reporting a median time of 5.3 min in post-operative cardiac surgery patients.^17,18^ A recent study by Brehaut et al., which examined the use of clevidipine in hypertensive acute stroke patients, reported a median 15 min from initial dose to goal BP.^27^ In our study, the median time to achieve the target BP after initiating clevidipine in the intensive management group was 20 min, compared to 10 min in the standard group. The shorter time to achieve target SBP in the standard group is largely explained by the broader and less restrictive SBP range (90–160 mmHg), which inherently facilitates faster attainment of target values. Previous BP trials in ischaemic stroke have not explicitly focused on the median time to reach target BP. However, the time to achieve BP control can be a critical window to maximise brain salvage and functional recovery. Maintaining optimal cerebral perfusion pressure during the early post-thrombectomy period is essential, as both hypotension and hypertension can compromise the brain’s ability to recover.

The trend towards better outcome in the standard treatment group is likely multifactorial. First, our study only included patients with successful reperfusion (≥TICI 2c). This is dissimilar to previous trials which included patients with successful recanalisation (≥TICI 2b) and may have enhanced the effect size for the standard treatment group due to residual distal occlusions. Hence, the present study sets the platform for discussing microemboli. Despite successful reperfusion, the arterial bed experiences increased resistance and, therefore, requires higher systemic arterial pressure (ie, standard arm) to maintain cerebral perfusion. Second, cerebral dysregulation limits hypotension tolerability. This is highlighted in ENCHANTED2/MT where median onset-to-reperfusion times were beyond 6 h, with poor outcomes demonstrated in the intensive treatment group.^11^

While maintaining appropriate cerebral perfusion improves penumbra viability, there is a concordant risk of haemorrhagic complications when overwhelming the endothelium. In fact, both inadequate perfusion and elevated arterial pressure can result in haemorrhage. In CLEVER, sICH rates were similar to those seen in other RCTs,^14^ and these data suggest that avoiding hypotension (SBP < 90 mmHg) and excessive hypertension (SBP > 160 mmHg) partially limits sICH rates. The low sICH rate in CLEVER may support the use of clevidipine to avoid excessive BP fluctuations in post-MT BP management; however, this rate may have been influenced by other factors such as sample size, heterogeneous BP targets or patient selection factors. There was a single patient with sICH within the standard treatment group which developed beyond the 24-h post-procedural period and was attributed to early anticoagulation therapy. Despite this complication, the patient’s discharge NIHSS was 2- and 90-day follow-up mRS was 2.

Although overall adverse event rates were low in CLEVER, we observed a higher frequency of AKI in the intensive BP control group. This may reflect the potential for renal hypoperfusion resulting from lower systemic pressures, particularly in the context of impaired autoregulation or suboptimal intravascular volume. Clevidipine’s potent vasodilatory effect could contribute to reduced effective circulating volume in this setting. While the study was not powered to assess renal outcomes, this observation raises important safety considerations that merit further investigation in larger, multicentre trials.

## Limitations

This study has several limitations. First, as the sample size in CLEVER is small with no sample size calculation used for the study, caution should be used when interpreting results; however, we retained clinical equipoise by demonstrating a clear BP dichotomy within the first 24-h post-procedural time window. Second, our population is predominantly a western population and may not be generalisable to the other ethnicities in which underlying stroke mechanism varies. Third, this study was not powered to detect significance of safety end points. Specifically, in order to detect a 2.5% difference in rates of sICH would require > 600 patients. Further studies could be considered, however, as suggested in our single patient who developed sICH, outcomes may not be clinically relevant. Fourth, our study did not employ a standardised approach to monitoring or controlling BPV, and no predefined thresholds were used to detect rapid BP changes. This may have contributed to variability in BP control, especially in the intensive group, and should be considered a methodological limitation of the trial. Lastly, CLEVER completed enrollment prior to the results of several RCTs^10,11,13^ on post-MT BP control, all of which demonstrated significantly lower function independence in patients with intensive BP targets. Therefore, the study design and rationale of CLEVER must be considered in the temporal context of its conception and enrollment period.

## Conclusion

In the randomised CLEVER trial, intensive BP control using clevidipine after MT failed to reduce the rate of haemorrhagic conversion or sICH and resulted in a numerically lower rate of good clinical outcome compared to standard BP control. Clevidipine was well tolerated in both cohorts and demonstrated a similar safety profile. Larger studies are needed to understand the efficacy and safety of clevidipine for BP control and the optimal BP threshold after MT.

## Supplementary Material

aakag005_11_9_25_Supplemental_Data
