# Supplementary material for: Clevidipine infusion for blood pressure management after successful revascularisation in acute ischaemic stroke: the CLEVER study
Source: Eur Stroke J. 2026 Feb 17;11(2):aakag005. doi: 10.1093/esj/aakag005 (PMC12911921; doi:10.1093/esj/aakag005)
Supplement: aakag005_11_9_25_Supplemental_Data [file aakag005_11_9_25_supplemental_data.docx]

**Supplemental Table I. CLEVER Trial Inclusion and Exclusion Criteria.**

| **Inclusion Criteria:** | 1. Age 18 or older  2. Acute hypertension (systolic blood pressure of greater than 130 mmHg) at recanalization  3. Anterior circulation ischemic stroke symptoms and confirmed occlusion (ICA, M1, or M2) on angiogram with mechanical thrombectomy initiated within 24 hours since last known well  4. Successful revascularization score of mTICI 2c or higher after mechanical thrombectomy  5. ASPECTS score of greater than 6  6. Premorbid mRS 0-4  7. Signed informed consent within 30 minutes from end of MT procedure. |
| --- | --- |
| **Exclusion Criteria:** | 1. Presence of any hemorrhage and/or ASPECT score ≤6 on baseline head CT scan  2. Pregnant or lactating  3. Acute traumatic brain injury  4. Patient on active dialysis  5. Intracranial neoplasm  6. Acute or recent STEMI in the last 30 days  7. Severe arrhythmias, unstable cardiac function  a. Patients who require vasopressor support are excluded from the study  *For example*: Dopamine, Dobutamine, Neo-Synephrine, Epinephrine  b. Patients requiring external cardiac pacing are excluded from the study  c. Patients with elevated troponin levels should be enrolled at investigator  discretion  8. Any terminal medical condition with life expectancy less than 6 months  9. Concurrent enrollment in another trial that could confound the results of this study  10. Intracranial Atherosclerosis:  a. culprit lesion or diffuse moderate to severe |

**Supplemental Table II. Sensitivity Analysis.**

|  | OR | Lower CI | Upper CI | p value | interaction term |
| --- | --- | --- | --- | --- | --- |
| AFIB (+) | 3.97 | 1.01 | 18.20 | 0.06 | 0.13 |
| AFIB (-) | 0.92 | 0.27 | 3.13 | 0.90 |  |
| M1 | 1.56 | 0.39 | 6.46 | 0.53 | 0.96 |
| M2 | 0.73 | 0.09 | 4.68 | 0.75 |  |
| ICA T | 2.13 | 0.33 | 15.20 | 0.43 |  |
| ASPECTS ≥ 8 | 1.71 | 0.69 | 4.37 | 0.25 | 0.99 |
| Age ≥ 65 | 2.60 | 0.89 | 7.89 | 0.08 | 0.16 |
| Age < 65 | 0.58 | 0.09 | 3.51 | 0.56 |  |
| HTN | 1.65 | 0.69 | 4.05 | 0.27 | 0.79 |
| HLD | 2.11 | 0.75 | 6.11 | 0.16 | 0.35 |
| Early Time Window (< 6 hours) | 2.00 | 0.61 | 6.76 | 0.26 | 0.74 |
| Late Time Window (> 6 hours) | 1.47 | 0.38 | 5.92 | 0.58 |  |
| TICI 2b | 0.44 | 0.02 | 4.46 | 0.52 | 0.60 |
| TICI 2c | 2.40 | 0.38 | 16.90 | 0.36 |  |
| TICI 3 | 1.35 | 0.38 | 4.86 | 0.64 |  |

**Supplemental Table III. Excursions (time/magnitude outside limits) in the CLEVER Trial.**

| AUC_SBP_, mm Hg $\times$ min / h | Standard group | Intensive group |
| --- | --- | --- |
| Mean $\pm$ SD | 0.83 (3.2) | 0.08 (0.70) |
| Median (IQR) | 0 (0, 0) | 0 (0, 0) |
| Min, Max | 0, 40 | 0, 15 |

**A.**

**
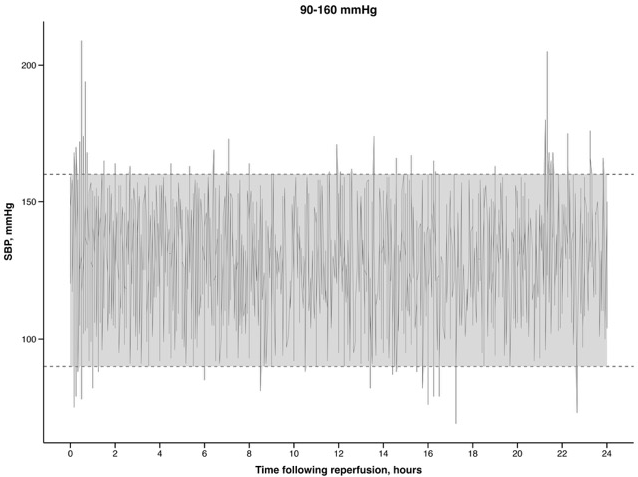
**

**
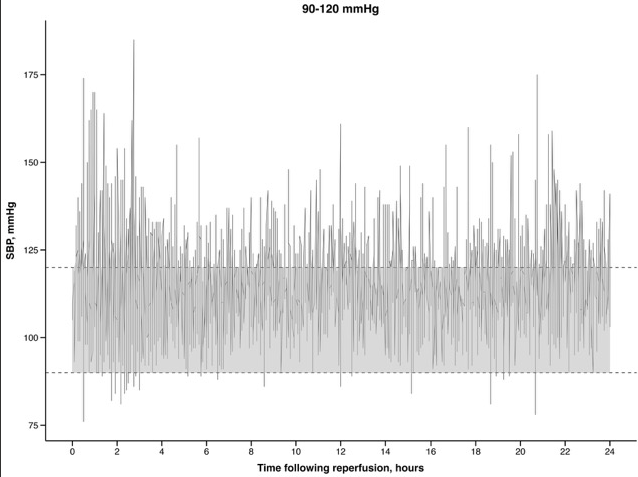
B**

**Supplemental Figure I. Mean SBP over the initial 24-hours in the A.) Standard BP and B.) Intensive BP management cohorts.**

**
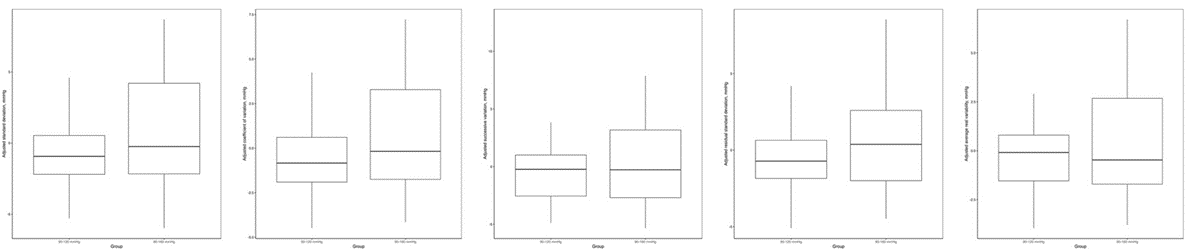
**

**Supplemental Figure II. Adjusted SBP standard deviation, coefficient of variation, successive variation, residual standard deviation, and average real variability between treatment groups.** The mean SBP SD, COV, SV, rSD, and ARV in the standard BP group were 13.1, 10.2, 14.0, 12.1, and 10.2 mmHg, respectively; while the values in the intensive BP target group were 10.7, 9.3, 11.9, 10.0, and 8.9 mm Hg, respectively. Adjusted SBP SD, COV, SV, rSD, and ARV did not significantly differ between the two groups.


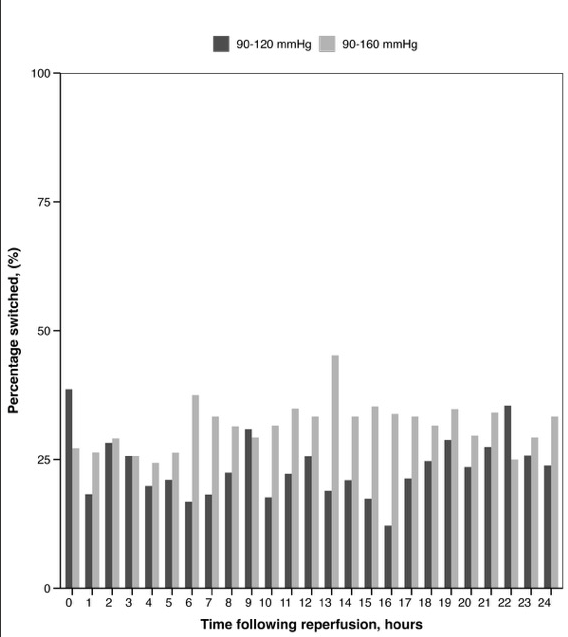


**Supplemental Figure III. Percentage of CLEVER patients that switched cohorts over 24hrs following reperfusion.**
